# Mandibular Distraction Osteogenesis: Upper Airway Management in Pierre Robin Sequence

**Published:** 2015-09-04

**Authors:** Patrick A. Newbury, Nicholas S. Adams, John A. Girotto

**Affiliations:** ^a^Michigan State University College of Human Medicine, Grand Rapids, Mich; ^b^Grand Rapids Medical Education Partners Plastic and Reconstructive Surgery Residency, Grand Rapids, Mich; ^c^Helen DeVos Children's Hospital Pediatric Plastic and Craniofacial Surgery, Grand Rapids, Mich

**Keywords:** mandibular distraction osteogenesis, Pierre Robin sequence, micrognathia, tracheostomy, airway obstruction

## DESCRIPTION

A 2-year-old boy born with Pierre Robin sequence (PRS) and CHARGE syndrome presented for airway management and removal of tracheostomy. Tracheostomy was placed for airway obstruction at an outside hospital shortly after birth. Mandibular distraction osteogenesis (MDO) using internal distractors was planned in an effort for early decannulation.

## QUESTIONS

1. **What is Pierre Robin sequence and what impact does it have on the upper airway?**
2. **What operative and nonoperative options are available to treat upper airway obstruction caused by Pierre Robin sequence?**
3. **Describe the physiology of distraction osteogenesis?**
4. **What are the indications, surgical options, and possible complications of mandibular distraction osteogenesis?**

## DISCUSSION

The clinical triad of retrognathia, glossoptosis, and airway obstruction defines PRS. Since its description by Dr Pierre Robin in 1934, many theories on the etiopathogenesis of PRS have been proposed; however, the mechanism remains unclear.^1^ Hypoplastic anterior growth of the mandible leads to posterior displacement of the tongue and varying degrees of airway obstruction. In severe cases, poor feeding, hypoxemia, and hypercarbia can lead to long-term sequelae such as failure to thrive and cor pulmonale. Cleft palate is present in up to 90% of PRS cases and is likely due to the high-riding tongue interfering with lateral palatine process descent and failure of fusion.^2^^,^^3^

Management of PRS requires a multidisciplinary approach. Nonsurgical interventions are sufficient in most PRS neonates and include prone positioning, supplemental oxygen, and continuous positive airway pressure. These measures are only temporizing in severe cases. Surgery remains the definitive option in patients with airway obstruction refractory to conservative treatment. Tongue-lip adhesion, tracheostomy, and MDO encompass the majority of surgical procedures. These are needed in less than 10% of nonsyndromic PRS patients.^4^ Tracheostomy establishes an immediate, definitive airway and historically has been considered the gold standard. However, it is associated with significant morbidity, high costs, and negative psychosocial impact.^2^^,^^4^ PRS patients requiring tracheostomy often rely on it for 2 to 4 years, which can lead to laryngeal stenosis and formation of granulation tissue.^3^ Since described in 1992, treatment of PRS with MDO has grown in popularity as an effort to avoid tracheostomy.^5^ It has since been shown to be a safe and effective treatment option for PRS-related airway obstruction.^2^ In addition to lower complications, MDO has been shown to carry a significantly lower cost than tracheostomy.^6^ MDO has also been shown to allow for early decannulation for patients with previously placed tracheostomy.^7^

Distraction osteogenesis is a method of elongating bone by progressive stretching of divided segments. It takes advantage of the normal healing process between 2 bone segments. In MDO, boney elongation and the soft-tissue envelope are simultaneously expanded, translating into anterior displacement of the tongue base. This mechanism leads to improved airway patency. After appropriate osteotomies have been made, distraction osteogenesis is divided into 3 phases. *Lag phase* is the time between osteotomy and the initiation of distraction, lasting 24 to 72 hours. The *distraction phase* consists of the daily elongation of the distraction device that spans the bone segments. The strain on the soft callus stretches the distance between the 2 bone edges. The tension force leads to increased metabolic activity and intramembranous ossification. Rates of 1 to 2 mm per day via 2 to 4 sessions are common. As desired length is achieved, distraction is concluded and ossification completes. This is termed the *consolidation phase* and lasts 6 to 12 weeks, concluding with device removal. MDO is indicated when conservative interventions fail to improve airway patency or in efforts of tracheostomy decannulation. Many techniques have been described, each with its benefits and drawbacks. The distraction device may be internally or externally placed. A mechanical saw is used to perform a carefully placed corticotomy, followed by osteotomy completion with an osteotome. This method allows for protection and preservation of the inferior alveolar nerve. For internal distractors, an extension arm penetrates the skin, allowing adjustment of the device. This may travel anteriorly to emerge near the chin or posteriorly emerging behind the ear.

Complications associated with MDO are as high as 35%, with a majority consisting of minor issues not requiring hospitalization. These include surgical site infection and poor scar aesthetics. A small percentage of patients require tracheostomy following distraction. Other less common complications include facial or inferior alveolar nerve injury during dissection and osteotomy formation, temporomandibular joint ankylosis, injury to unerupted dentition, and asymmetry.^2^^,^^8^

MDO is a safe and effective method to increase airway patency in PRS. This case underscores the role of MDO in decannulation for PRS patients with previous tracheostomy placement. MDO can be considered an effective option for early decannulation in PRS patients.

## Figures and Tables

**Figure 1.**
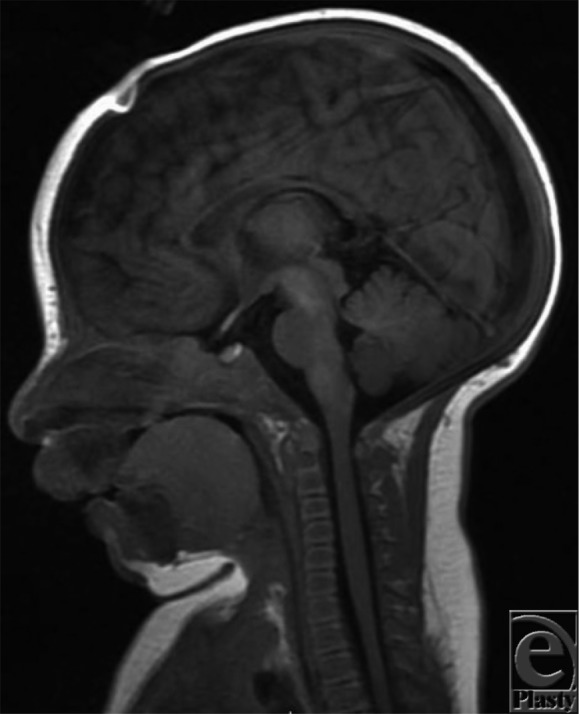
Magnetic resonance imaging, sagittal T1 FLAIR image at 2 months of age, indicating posterior displacement of the tongue causing significant pharyngeal airway obstruction.

**Figure 2.**
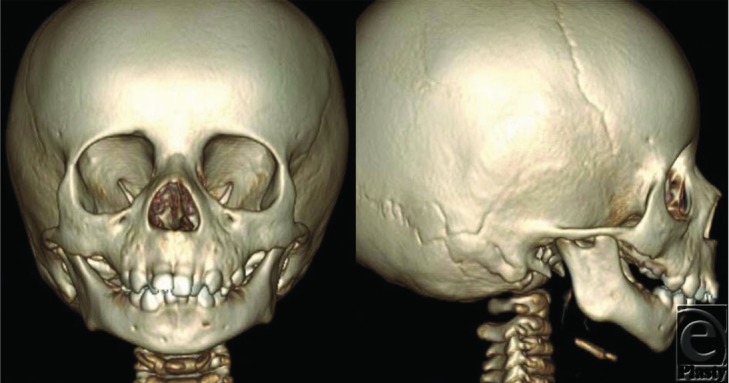
Preoperative 3-dimensional reconstructed maxillofacial computed tomographic scan.

**Figure 3.**
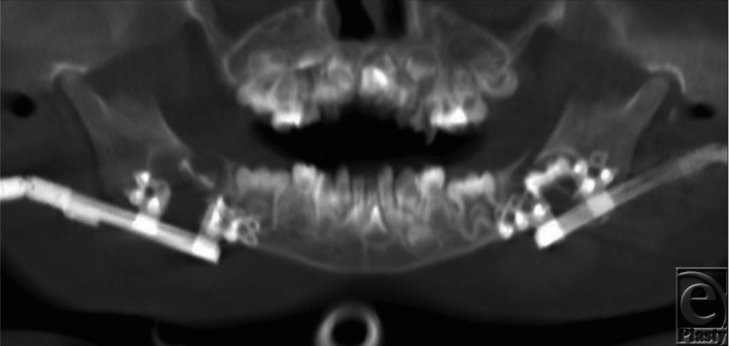
Panorex image taken during the distraction phase, with the distractor arms directed posteriorly.

**Figure 4.**
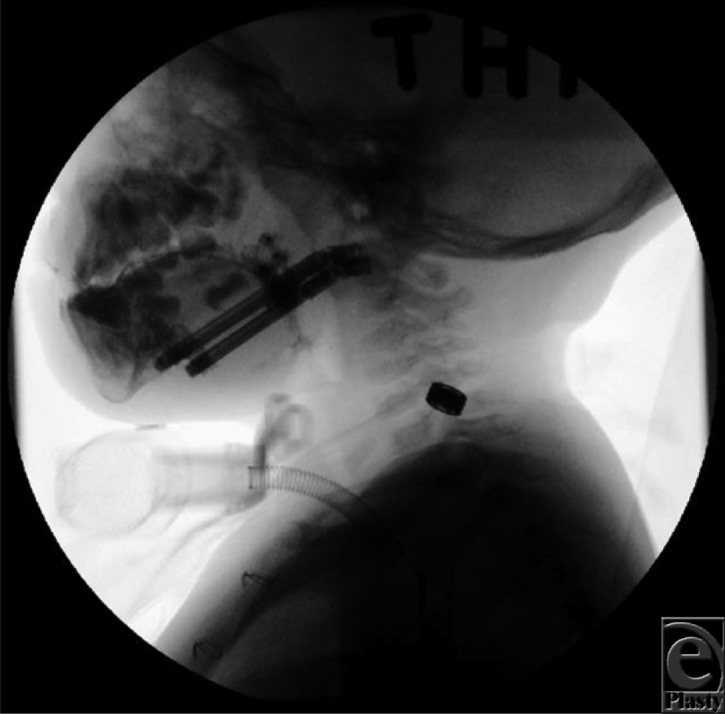
Fluoroscopy image taken during the consolidation phase prior to removal of distraction devices and tracheostomy decannulation.
